# Calibration of Partial Safety Factors of Sample Masonry Structures

**DOI:** 10.3390/ma14175003

**Published:** 2021-09-01

**Authors:** Joanna Zięba, Izabela Skrzypczak, Lidia Buda-Ożóg

**Affiliations:** 1 Department of Building Structures, Faculty of Civil and Environmental Engineering and Architecture, Rzeszow University of Technology, Poznańska 2, 35-084 Rzeszow, Poland; lida@prz.edu.pl; 2 Department of Geodesy and Geotechnics, Faculty of Civil and Environmental Engineering and Architecture, Rzeszow University of Technology, Poznańska 2, 35-084 Rzeszow, Poland; izas@prz.edu.pl

**Keywords:** masonry constructions, partial factors, compressive strength, mortar, masonry units, probabilistic analysis, coefficient of variation

## Abstract

Technological progress in masonry structures has resulted in the creation of competitive solutions, which force the need for an ever deeper recognition of this type of structure. Masonry is a composite with heterogeneous strength properties. Therefore, the most appropriate way to accurately describe the behavior of the masonry structure under the influence of the working load are experimental research and their statistical and probabilistic analysis. This article presents a series of experimental tests carried out on real masonry structures. The results of the experiments were subjected to static evaluation, determining the most important parameter in the probabilistic analysis—the coefficient of variability of strength. The variability obtained in the experimental studies was used to determine the safety of the structure in the probabilistic method. Achieved values of coefficients of variation and safety coefficients proved to be satisfactory and adequate to the emerging technological progress in the production and embedding of masonry components.

## 1. Introduction to the Design of Masonry Structures

### 1.1. The Issue of Masonry Structures

The dynamic development of technologies and building materials has not resulted in a departure from traditional, classic methods of building structures. The traditional method of erecting structures made of masonry components has long dominated the Polish and global construction market, in particular in the residential segment, but also in the construction of public utility facilities. The oldest type of masonry element is dried brick. For its production, materials available in the region were used—clay, loam, or silt. Fired brick began to be used only around the 4th century BC, then it became the most important building material. Initially, brick was treated as a valuable decorative and architectural material. In the Renaissance and Baroque, however, it lost its decorative value. Since then, it has been treated only as an element of a structure with imposed load-bearing and quality requirements in relation to the component itself, structural elements, as well as the implemented construction objects.

Masonry is a construction material made of masonry elements arranged in a certain way connected permanently with a mortar. Significant variations in materials, technology, and performance mean that masonry structures are much more difficult to be diagnosed than concrete or reinforced concrete [1]. What should be emphasized is that masonry is a typical material with anisotropic properties. Good quality components do not imply good wall masonry. The cooperation of masonry units and joint filling mortar in the structure depends on the type and direction of load application in relation to the support joints. Currently, we have access to many masonry manufacturers on the market. Technological progress has resulted in the creation of competitive solutions in both execution and design, which forces the need for an ever deeper recognition of this type of structure. The market offer of masonry units and mortars has changed a lot in recent years.

**Figure 1:**
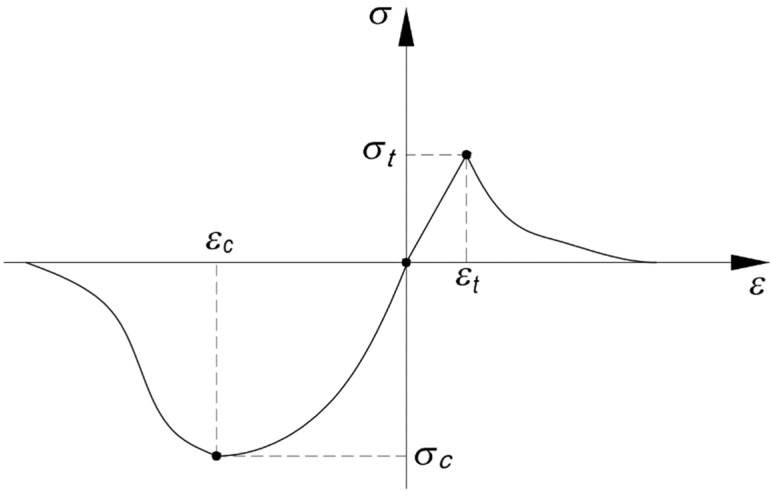
Graph of stress σ-strain ε at uniaxial compression of quasi-brittle material [1].

The masonry structure should be considered as a heterogeneous material consisting of various types of masonry units connected in a proper way with mortar. The load-bearing capacity of the masonry is determined by the strength, deformability of its components, as well as the features of the masonry composite itself, such as: bonding of units, wall thickness, the presence of longitudinal joints, and the specificity of the acting load. The ratio of low tensile strength and high compressive strength is the basis for the classification of masonry as a quasi-brittle material. The process of destruction of this type of materials occurs as a result of the progressive development of internal micro-scratches, which at a later stage of the load usually turn into visible macro-scratches. For brittle materials, it can be assumed that the dependence of stresses on deformations has a linear course up to a value of 33% compressive strength and tensile strength [2,3]. After exceeding the limit of tensile or compressive strength, constitutive compounds for the masonry and its components are described by non-linear relationships—Figure 1. Therefore, the most appropriate way to accurately describe the behavior the masonry structure under the influence of the acting load is experimental research.

The PN-EN 1996-1-1 standard [4] recommends that the characteristic compressive strength of masonry should be determined each time in experimental tests. The designer uses a simplified approach based on the assumption that the wall is a homogeneous material. For this purpose, the analytical dependencies determining the masonry strength given in the PN-EN 1996-1-1 standard [4] are used:

(1) $$f_{k}=Kf_{b}^{\alpha }f_{m}^{\beta }$$

where: *Κ*, *α*, *β*—coefficients determined empirically; $f_{b}$—compressive strength of clay masonry units; $f_{m}$—compressive strength of mortar.

The calibration of the *K* coefficient values was presented in the research of Edgel, Bright, and Heath [5], among others. In the paper [6], on the basis of his own research, Schubert proposed the values of *Κ*, *α*, *β*.

The polish annex of the PN-EN 1996-1-1 [4] standard introduces precise values of empirical coefficients and proposes to present the general formula in four variants, which are summarized in the table—Table 1.

The characteristic resistance of masonry is closely related to the geometry of the masonry units used, therefore, the calculations should take into account that the masonry component belongs to the appropriate group. Geometric requirements for individual groups of masonry units are included in PN-EN 1996-1-1 [4]. The group of masonry units is determined on the basis of:

- volume of all holes (percentage in gross volume),
- single hole volume (percentage in gross volume),
- declared thickness of internal and external walls,
- declared equivalent thickness of internal and external walls (% of gross width).

Due to the presented geometric parameters, four groups of masonry units are distinguished:

- group 1—masonry units made of autoclaved aerated concrete (AAC), natural and artificial stone
- group 2 and 3—vertically hollow masonry units
- group 4—horizontally hollow masonry units

The group of masonry units is the basis for determining the *K* coefficient, which is the basis of almost every formula for the characteristic strength of a masonry. Accepted in the PN-B-03002: 2007 standard [7], the values of the *K* coefficient, in fact in most cases unchanged, are still valid in the PN-EN 1996-1-1 standard. When calibrating the value of the *K* coefficient, the results of research carried out by the Building Research Institute in 1999–2004 [8] were used. The values of the *K* coefficient recommended for calculations are included in the relevant tables of the main part of PN-EN 1996-1-1 [4] and the National Annex.

The last modification of the *K* coefficient was carried out in 2014 by introducing an amendment to PN-EN 1996-1-1+A1:2013-05/Ap2 [4]. The research conducted at the Silesian University of Technology changed the value of the K coefficient in the power formula for calculating the characteristic compressive strength of a masonry made of silicate elements [9]. In the National Annex NA of the PN-EN 1996-1-1 [4], the value of the *K* coefficient for group 1 silicates and thin layer mortars was changed from 0.55 to 0.60.

The compressive strength of the wall depends on many factors, such as the shape and size of the test elements. The actual masonry structure usually has much larger dimensions than the tested standard test elements. A very significant influence of the shape and dimensions of the test piece on the obtained values of the compressive strength of the entire wall was proved in the works [10,11,12].

The analysis of compressed masonry structures has been presented in many domestic and foreign scientific publications [13,14,15]. There are also publications on the cyclic loading of masonry structures [16,17,18].

### 1.2. Partial Safety Factors in Masonry Structures

The basis for designing various types of structures are the appropriate design values of material properties and actions. Recommendations in the standards regarding: material properties, structural calculation models, method of determining the cooperation of different materials, load summation are burdened with some uncertainty. Therefore, when designing all types of structures, security measures are taken into account that the standard procedures are not sufficiently compatible. These measures, both in masonry and any other construction, apply in the form of partial factors used on the load side and on the capacity side.

The design strength of the masonry is determined from the relationship [19]:

(2) $$f_{d}=\frac{f_{k}}{\gamma_{M\;}}$$

(3) $$\gamma_{M}=\gamma_{m}\cdot \gamma_{Rd}$$

where: $\gamma_{m\;}$—partial factor for materials, including uncertainties about geometry and modelling; $\gamma_{Rd}$—partial factor taking into account the uncertainty of the theoretical calculation model of the structure.

Since the masonry is a structural element burdened with many unknowns, determining the $\gamma_{m\;}$ value for the masonry is particularly difficult and also burdened with high uncertainty. 

How to determine analytically the values of $\gamma_{Rd}$, $\gamma_{m}$—or directly $\gamma_{M}$ Eurocodes— unfortunately, are not specified. The recommendation of standards is to set these values in accordance with the scope of application of the test results. Partial factors are values from the NDP group—“nationally determined parameters”—reserved for determination by standard national organizations in consultation with the competent national authorities of EU Member States. The task is difficult because it connects with the level of construction safety required in the country. Values of the partial factor used in calculations of masonry structures are specified in National Annexes to PN-EN 1996-1-1 [4]. 

Masonry units should be primarily assigned to the appropriate category. The category of masonry units is directly related to the control of their production. For mortars, from the point of view of a partial factor, it is important to divide into designed mortars (composition specified by the manufacturer) and prescribed mortars (manufactured on site). PN-B-03002:1999 [20] also introduced the concept of the class of execution of works. The current EN 1996-1-1 [3] standard distinguishes between two classes of execution of works: class A and class B. The design designer decides about the class of masonry works.

In the original PN-87/B-03002 [21] standard, the values of the $\gamma_{m}$ partial factor depended solely on the type of masonry units (solid, hollow elements) and the working conditions under load (compression, shear). The values of the coefficient were then in the range of 1.5–1.9. Additionally, a coefficient increasing $\gamma_{m}$ was also introduced, marked as $m_{m}$, which depended on the properties of the mortar used. The values of $\gamma_{m}$ proposed in the PN-B-03002:1999 [20] standard are presented in the table—Table 2. This table in an extended version was presented in successive versions of the applicable standards.

The values of $\gamma_{m}\;$adopted in the PN-B-03002:2007 [7] standard are the values recommended also in the currently applicable provisions of PN-EN 1996-1-1 [4]. The analogy of the partial factor value in the PN-B-03002:2007 [7] and PN-EN 1996-1-1/NA [4] standards clearly show the lack of differences in the approach to the safety of masonry structures at the turn of several years of development of scientific works.

**Figure 2:**
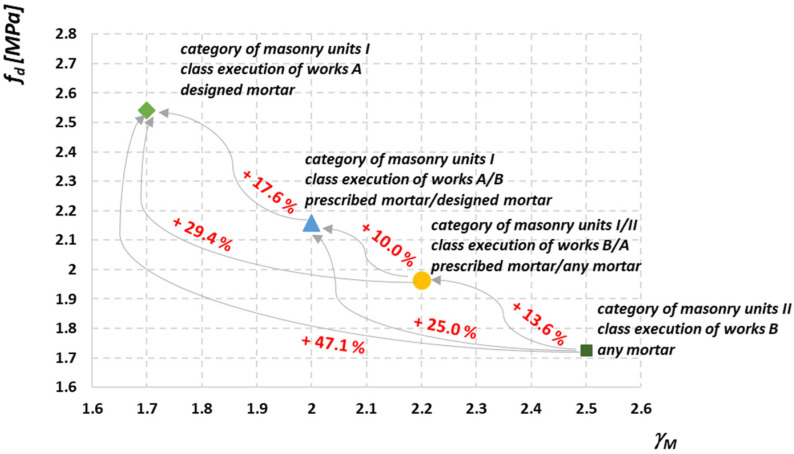
Differentiation of the compressive strength of an exemplary masonry structure for different configurations of the masonry quality [22].

Figure 2 shows the percentage differences in the compressive strength of an exemplary masonry structure for different variants of the configuration of the category of elements with mortar and the class of execution of works. Significant differences between extreme approaches to the design of masonry structures indicate a very high importance of parameters determining the quality of masonry in the computational strength. While the quality of the masonry components is determined by the manufacturer, the main role of the designer is to choose the appropriate class of wall execution. After the author conducts an environmental interview, it turns out that in practice the vast majority of designers choose class B, due to the greater value of the partial factor, and therefore greater safety of the designed structure, in favor of the designer. However, the question arises whether the introduction of two columns of the partial factor value in the Polish National Annex to PN-EN 1996-1-1 [4] is necessary.

Table 3 shows the values of the partial factor presented in the main part of PN-EN 1996-1-1 [4]. The values in bold in the second and third columns correspond to the values of the partial factor adopted in the Polish National Annex PN-EN 1996-1-1 [4].

Referring to the provision that the values of the wall strength are specified in national annexes, there are publications that present the analysis of the computational strength of the masonry on a European scale [24]. The differentiation of the value of $\gamma_{M}$ depending on the class of execution of works was adopted in their National Annexes: France, Hungary, Great Britain. The other countries adopted one work performance class in their recommendations, which significantly facilitates the process of designing masonry structures. The analyses carried out in the publication showed that the recommendations adopted in the Polish National Annex to PN-EN 1996-1-1 [4] allow for obtaining relatively high values of the computational strength of the wall. The example diagram shows the dependence of the design strength of the masonry $f_{d}$ on the strength value of its component, the masonry unit $f_{b}$. The design strength of the masonry was calculated in accordance with the recommendations of the annexes to PN-EN 1996-1-1 [4] of each of the individual countries, taking into account the appropriate formulas and coefficients.

**Figure 3:**
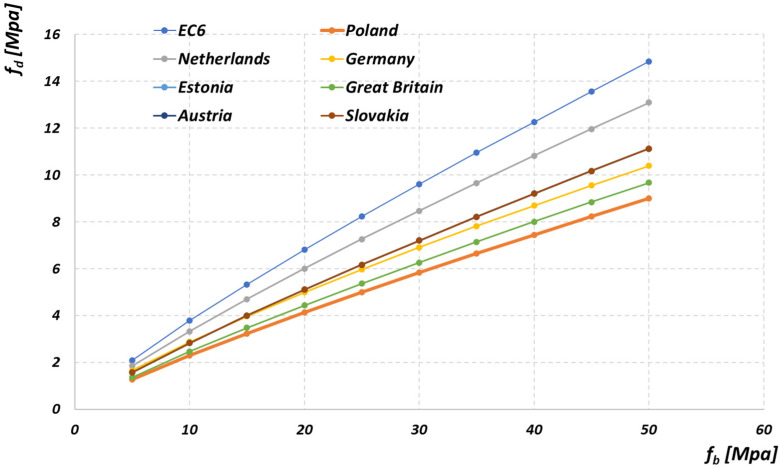
Dependence of the design strength of the masonry on the strength value of the masonry units for the masonry made of silicate units on thin layer mortar.

The most conservative approach to the design of masonry structures in Poland applies to silicate elements—Figure 3. In the case of clay units and autoclaved aerated concrete units, the differences in the calculated strengths of the masonry for individual countries are smaller, but in many cases still controversial. The presented considerations may be an argument for the need to conduct advanced research of masonry structures in Poland, both experimental and analytical, which will allow for a more accurate verification of the strength parameters of the wall, and as a result obtaining the results of calculations of masonry structures corresponding to the top EU countries.

## 2. The Idea of a Probabilistic Approach to Structure Design

The way in which the structure will behave under the appropriate type and size of load is strictly dependent on the strength of the materials and the stiffness of the structure. In turn, whether the response of the structure is satisfactory for its designer or user, depends on all the requirements that the structure should meet. Variations in design parameters must therefore necessarily be included in the consideration of the safety and reliability of the structure.

Safety and reliability are fundamental concepts in the design, construction, and operation of structures. Reliability in general is the ability of a structure to perform its designed function over a specified period of operation. Both the structure and the environmental impact, as well as the criteria for assessing the quality of the structure (the ability to perform the given functions) are random and may change over time. A consequence of this is that the measure of reliability is the probability that the structure will not exceed certain limit states during the assumed service life. Safety in the general sense means no threat to human life and health as well as economic, social, and ecological losses during the designed period of use [25].

When designing any structure, including masonry, you should be aware that almost all factors are uncertain and nothing can be predicted with absolute certainty. This basic fact leads to the idea of probabilistic and stochastic treatment of problems in every possible field. In civil engineering, most problems are solved by a deterministic approach that displaces difficult and complex stochastic solutions [23]. A number of simplified methods have also been developed in an attempt to combine deterministic and stochastic approaches, such as the semi-probabilistic concept of construction safety that underlies standard recommendations. For the 2nd level probabilistic method—the FORM (First Order Reliability Method) method—the relationships between the values of reliability index *β* and the values of partial safety factors were formulated. The reliability index *β* is the most commonly used, popular measure of security in semi-probabilistic methods. In the simplest case, when two uncorrelated basic variables are considered in the limit state equations: random load capacity *R* and random effect of *E* actions, the interpretation of the index *β* can be presented as the distance of the straight limit states from the beginning of the coordinate system representing the expected state of the structure. In the case of a non-linear condition of structural safety, the reliability index is defined as the minimum distance from the origin of the coordinate system to the hyperspace determining the limit state of the structure.

Determining the reliability index β is associated with the ordinances regarding the reliability of construction works, given in Annex B of the PN-EN 1990 [26] standard. In order to differentiate the reliability, consequence classes (CC) have been established, which in the PN-EN 1990 [26] standard have been defined as CC1, CC2, CC3, specifying respectively: low threat to human life, average threat to human life, and high threat to human life. The defined consequence classes CC correspond to the reliability classes (RC): RC1–RC3. Recommended minimum values of reliability index β, related to reliability classes, are also given in PN-EN 1990 [26]—Table 4.

The index values corresponding to the RC2 reliability class are significant, as it is in relation to this class that the safety of the structure is ensured using the set of applicable Eurocodes. Generally speaking, the use of a set of coefficients in force in Eurocodes determines ensuring structure reliability at the RC2 class level. Level II methods use some well-defined approximations and allow for results that, in most structural applications, can be considered accurate enough.

Since probabilistic calculations for assessing the reliability of structural elements and buildings are increasingly important, estimation of statistical parameters of material properties plays a major role. While the required information based on extensive test data exists for steel and concrete structures, there is still a lack of information on masonry structures, especially those made from modern materials. In the literature, we can find publications presenting probabilistic analyses of existing masonry structures [27,28].

An important parameter in the analysis of the results of experimental tests is the coefficient of variation ν, showing the level of differentiation of individual strength values from the average value obtained from tests, as well as determining the appropriate probability density distribution.

The coefficient of variation is a parameter widely used in statistics for determining the measure of variation in a characteristic. It belongs to the category of relative measures of variation. The coefficient of variation allows you to assess the strength of diversity of a given statistical population by showing the strength of the variable, and also evaluates the arithmetic mean. A high value of the coefficient indicates strong differentiation, and vice versa.

In sample publications [29,30,31,32,33], is presented the coefficients of variation in compressive strength of various types of masonry—Table 5.

**Figure 4:**
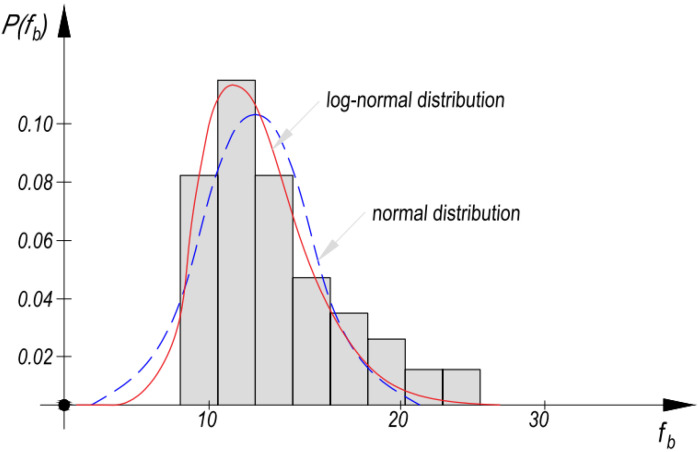
Sample histogram and matched density probability function of brick strength $f_{b}$.

Examples of Małyszko research [34] indicate that the results of masonry testing are adapted to the log-normal distribution of probability density (coefficient of variation 0.25)—Figure 4.

The assumption of the logarithmic—normal distribution of the probability density function for the compressive strength of masonry elements and the masonry themselves—is justified by numerous publications and recommendations [33,35,36,37,38]. Distribution of strength with clearly left-hand asymmetry was obtained in [39].

## 3. Case Study: Experimental Tests of Example Masonry Structures

As part of this article, an analysis of the results of experimental research carried out for the purposes of the PhD dissertation on selected masonry structures made of clay brick [40] and autoclaved aerated concrete blocks was made. The performed tests were destructive tests. It is worth mentioning here that non-destructive methods of analyzing the behavior of a masonry structure under load are becoming more and more popular [41,42,43].

Classic constructions were the subject of considerations: a brick pillar made of clay brick with ordinary cement-lime mortar, and a fragment of a masonry wall made of autoclaved aerated concrete blocks with thin mortar. This article will present the results of basic tests of masonry structures and associated tests, i.e., tests of selected components of masonry structures.

**Figure 5:**
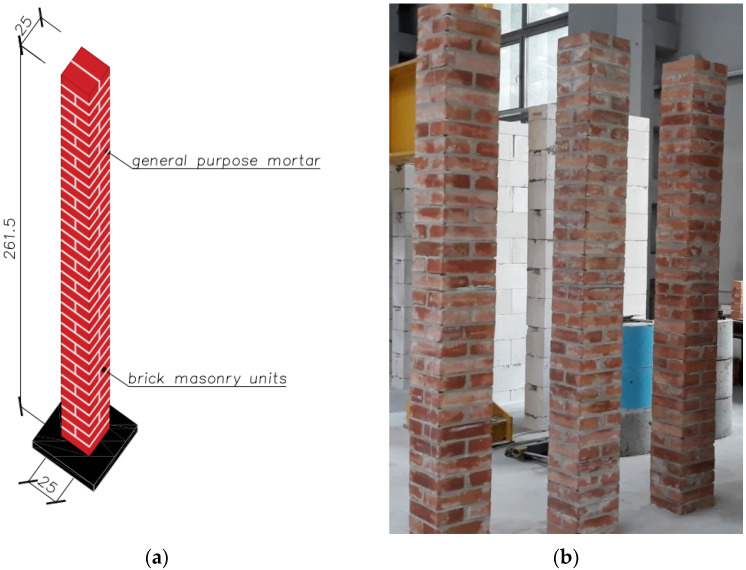
Shape and dimensions of the brick masonry pillar model in compression tests: (**a**) shape and dimensions of the pillar model (**b**) pillar models [23].

**Figure 6:**
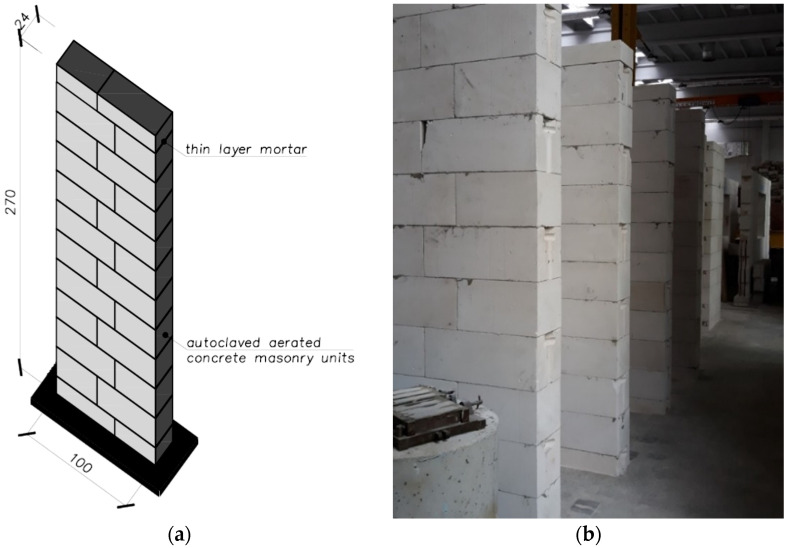
Shape and dimensions of the masonry wall model made of autoclaved aerated concrete units used in compression tests: (**a**) shape and dimensions of the masonry wall model, (**b**) masonry wall models made.

The program of basic tests included tests of compressive strength of selected masonry structures. Structural tests on a real scale were conducted in accordance with PN-EN 1052-1: 2000 [44]. The diversity of models concerned the shape (purpose), type of masonry units, and mortar used. The following research models of masonry structures were used in the tests of the main part:

- brick masonry pillars on M5 ordinary cement and lime mortar with dimensions of 250 × 250 mm and height of 2615 mm—six identical models were tested: five models under uniform load, one model tested cyclically—Figure 5;
- masonry walls made of autoclaved aerated concrete blocks on thin-layer mortar with dimensions of 240 × 1000 mm and height of 2700 mm—six identical models were tested—Figure 6.

### 3.1. Tests of Masonry Components

In order to obtain the value of the properties of materials used in the tests, in the first stage of experimental tests, mortar compressive and tensile strength tests were carried out, as well as masonry compressive tests, i.e., in this case only clay bricks.

**Figure 7:**
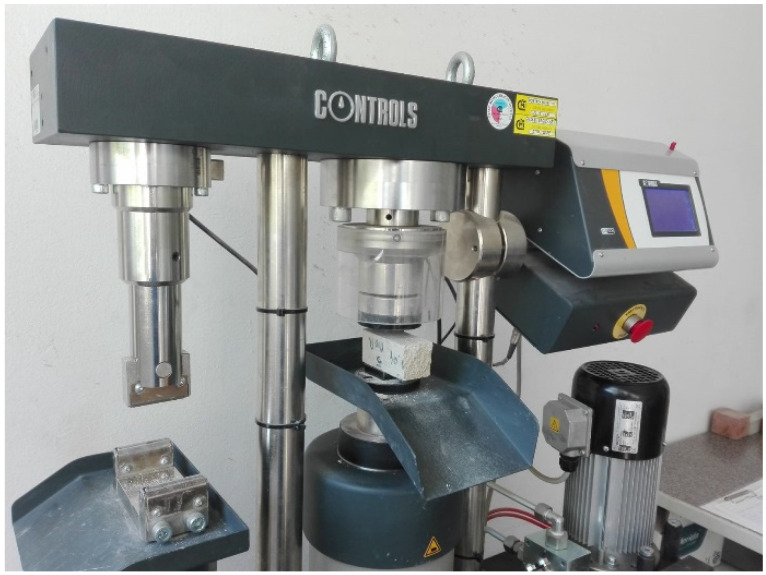
Mortar strength tests—a compression mortar test stand.

Laboratory tests of mortars were carried out in accordance with the requirements of PN-EN 1015-11: 2001 [45]. Samples obtained from ordinary cement-lime mortar for masonry of clay brick pillars and thin-layer mortar for masonry walls were tested. The compressive strength of the mortar was determined on boom fragments obtained as a result of bending tensile strength tests. The study was carried out at a special stand that allowed the concentrated load from the testing machine to be distributed over the surface of “beams”. The compressive strength testing machine had a range of 100 kN and automatic control of load growth continuously, without jumps—Figure 7 In the case of mortar, 36 samples of ordinary cement-lime mortar and 36 samples thin-layer mortar were compressed.

**Figure 8:**
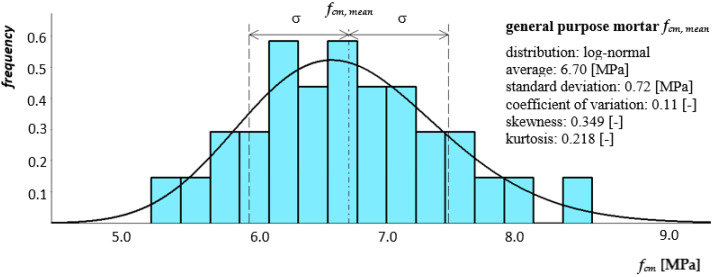
Histogram together with the approximate function of the compression strength distribution of general purpose mortar as a component of a clay brick masonry pillar.

**Figure 9:**
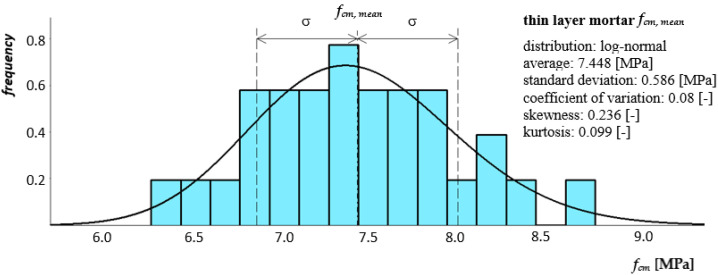
Histogram together with the approximate function of the compressive strength distribution of thin-layer mortar as a component of a masonry wall made of autoclaved aerated concrete blocks.

Figure 8 and Figure 9 show histograms together with the approximate density function of the compressive strength distribution of both mortars.

**Figure 10:**
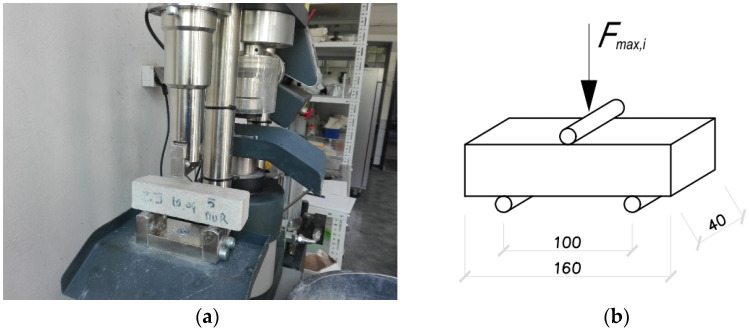
Mortar strength tests: (**a**) bending tensile testing stand, (**b**) bending tensile testing scheme.

**Figure 11:**
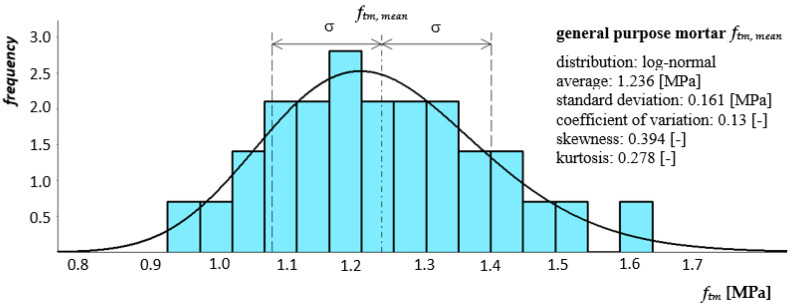
Histogram together with the approximate function of the tensile strength distribution when bending general purpose mortar as a component of a clay brick masonry pillar.

**Figure 12:**
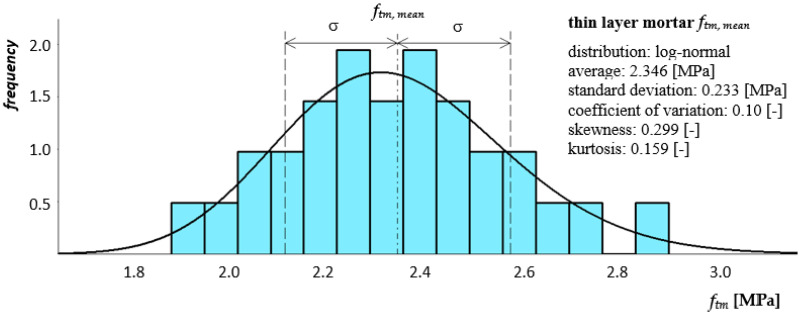
Histogram together with the approximate function of the tensile strength distribution when bending thin-layer mortar as a component of a masonry wall made of autoclaved aerated concrete blocks.

The principle of testing the mortar tensile strength when flexing was based on three-point loading of hardened, prismatic mortar samples (bars). In accordance with the requirements of the standard, a minimum of three 40 × 40 × 160 mm beams were formed from each earnings prepared for the construction of one pillar or masonry wall model. The samples were placed in a testing machine on two articulated sliding supports, in a diagram of a simply supported beam and loaded with concentrated force—Figure 10. Figure 11 and Figure 12 show histograms together with the approximate function of the density of the tensile strength distribution when bending both mortars. Eighteen samples of each mortar were used in the bending tensile tests.

**Figure 13:**
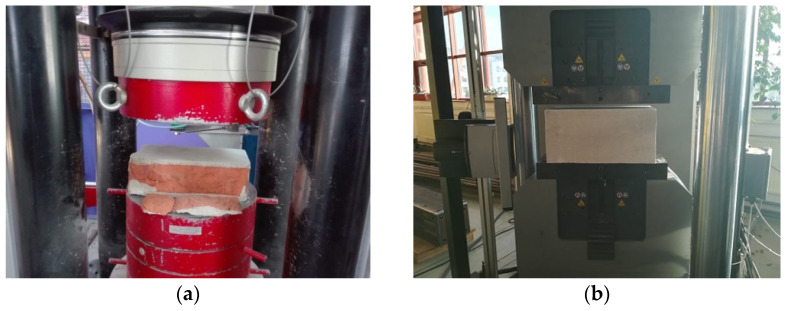
Strength tests of masonry units: (**a**) tests of solid bricks, (**b**) tests of autoclaved aerated concrete blocks.

**Figure 14:**
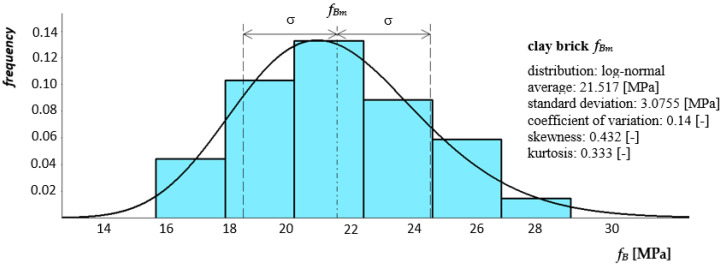
Histogram together with the approximate function of the compression strength distribution of clay brick.

**Figure 15:**
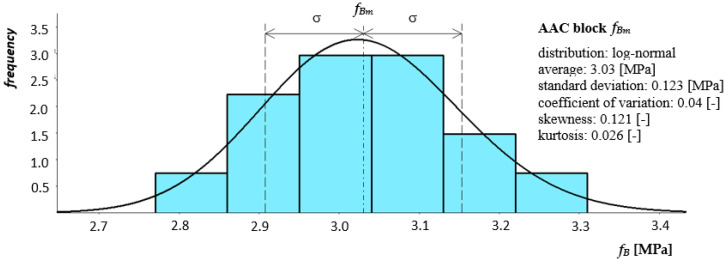
Histogram together with the approximate function of the compression strength distribution of autoclaved aerated concrete block.

In the analysis of the masonry structure, it is important to determine the strength parameters of masonry elements and their geometric characteristics. Compressive strength was the basic tested feature of masonry elements, which is justified because this parameter largely determines the strength of the wall. However, the comparison of, for example, the test results for bricks, makes it difficult to use in the past various test procedures, the shape of the samples, the method of preparing the base surface, the moisture level and many other factors significantly affecting the test result. Compressive strength tests of masonry units were carried out in accordance with the PN-EN 772-1: 2003 [46] standard in the air-dry state. Six whole 250 × 120 × 65 mm ceramic units and six autoclaved aerated concrete blocks with dimensions of 240 × 450 × 240 mm were tested. The elements were placed in a testing machine and loaded until destruction, reading the value of the destructive force. The view of the test stand is shown in Figure 13. Figure 14 and Figure 15 show the histogram with an approximate function of the density strength distribution of the tested bricks.

The results of accompanying experimental tests presented confirm the improvement in the quality of both mortar and modern masonry components. In the case of general purpose mortar, coefficients of variation with values of *ν* = 0.11 and *ν* = 0.13 were obtained for compressive strength and bending tensile, respectively. For thin-layer mortar, coefficients of variation were obtained with values *ν* = 0.08 and *ν* = 0.10. Higher values of the coefficient of variation were obtained for general purpose mortar, which is directly related to the production process. General purpose mortar is a mortar whose strength is obtained on the basis of the proportions of ingredients and is usually carried out at the destination. In turn, thin-layer mortar is a mortar with a specific composition produced by a specific manufacturer. In the case of clay brick, the variability of its most important feature, i.e., compressive strength, was determined at the level of *ν* = 0.14. For AAC block coefficient of variation was determined at the level of only *ν* = 0.04. Material variability presented on the basis of conducted tests is relatively small. The values of coefficients of variation are satisfactory given the large number of factors affecting the quality of the components of the masonry.

### 3.2. Basic Tests of Selected Masonry Structures

**Figure 16:**
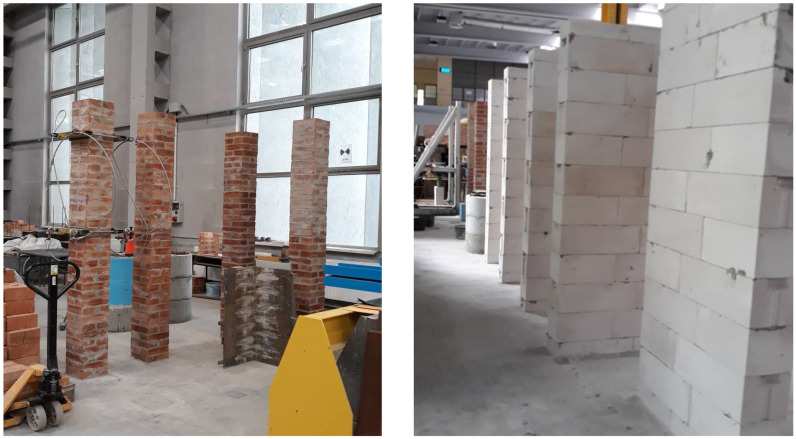
Photographs from the process of preparing models for research [23].

**Figure 17:**
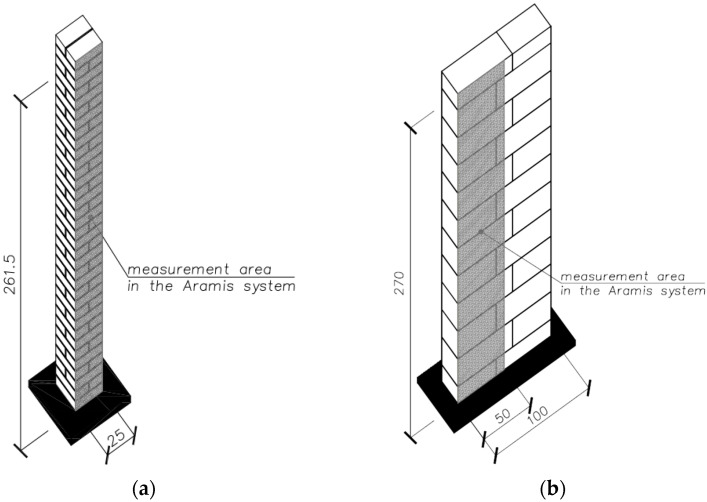
Measurement area in the Aramis system for (**a**) a model of clay brick pillars, (**b**) a masonry wall model made of autoclaved aerated concrete blocks.

All basic models of the analyzed masonry structures were tested in the DrBM-600 testing machine with manual control of load increase and indication accuracy 0.001 kN. The tests determined the compressive strength of the masonry perpendicular to the support joint on the basis of the results of the strength of the test models loaded up to destruction. In accordance with the recommendations of the standard, the materials and method of joining corresponded to those used in practice—Figure 16. In the case of model pillars, a leveling layer was needed. A modern system for three-dimensional deformation measurements Aramis 6M (GOM GmbH, Braunschweig, Germany) was used to measure deformations on the surface of samples [47]. Readings of displacements were made using cameras placed on a special tripod and arm. The Aramis system allows you to measure 3D displacements in a specific area. Aramis recognizes the surface structure of the measured object on the basis of photos. After recording all the photos, Aramis compares them with each other by assigning characteristic points to square or rectangular small surfaces called facets, and then finds these characteristic points on subsequent photos. It then calculates the displacement for the given point object. In the case of masonry tests using the Aramis system, proper surface preparation is necessary, i.e., creating a random pattern—Figure 17. It should be noted that the correct preparation of the sample surface and uniform lighting during the test has a huge impact on the correctness of the reading.

**Figure 18:**
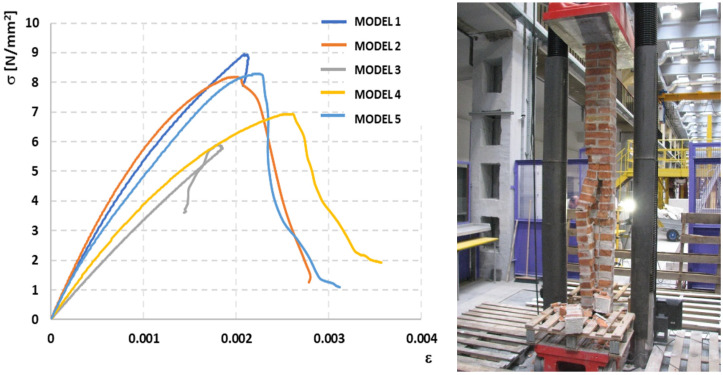
Stress-strain under compression relationship of clay brick pillars under uniform load and view of an example element after the test [23].

**Figure 19:**
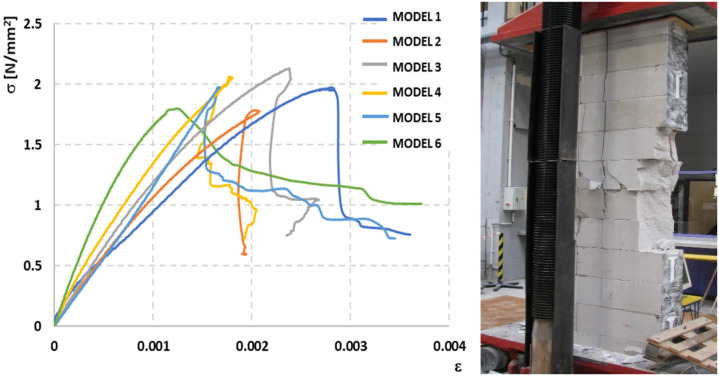
Stress-strain under compression relationship of masonry wall model made of autoclaved aerated concrete blocks under uniform load and view of an example element after the test.

Based on the measured values of horizontal and vertical displacements, diagrams of the *σ-ε* dependence were prepared, which were used to determine the modulus of elasticity of the masonry in the range of 0.00–0.33*σ_max_* and the Poisson’s ratio at the level of 0.33*σ_max_*—Figure 18 and Figure 19.

Statistical measures were determined for the individual results of the experimental studies—Table 6. For this purpose, two methods of statistical analysis were used: the classical method and the data evaluation resistance method. The application of the classical method required the elimination of doubtful results, hence the Q-Dixon test was used to evaluate the data.

Immune statistics methods provide less than the classical models in terms of impact of outliers and other data anomalies on the measurement result. For the data obtained from the research, an immunity method was used to evaluate the data with scaled median deviation. In this method, the median of all the results obtained should be determined, then the median deviation should be calculated and scaled by the multiplier value for random samples of the appropriate number [40].

As a result of statistical evaluation, two methods obtained similar mean and median values and relatively different standard deviations. Classic statistical methods of data processing are based on the assumption of modeling their dispersion by a known probability distribution, usually, mainly due to less tedious calculations, it is assumed that this is a normal distribution. Many scientists point to an unreasonable but widespread deep faith in the universality of normal distribution. In fact, in very few cases, e.g., when the measurement result is determined from a very large number of repeated measurements, the distribution of the results of individual measurements can be treated as fully normal. The standard deviation in the classical method is usually smaller than in the robust method of data evaluation. Methods of immunity statistics, due to low sensitivity to outliers, help improve the reliability of results, especially for samples with small numbers. The results of the conducted research and the resulting observations were an impulse to carry out further statistical analyzes.

## 4. Application of the Probabilistic Method to Determine the Level of Partial Safety Factors of Tested Structures

Based on the results of the experimental tests, the partial safety factor for the analyzed masonry structures, tested under compressive load, was determined. For this purpose, the calibration method recommended in the standard [26] and the literature [48,49,50,51].

Design load capacity according to the standard [26] can be expressed as (4):

(4) $$R_{d}=\frac{1}{\gamma_{Rd}}R\left(\eta X_{d};a_{d}\right)=\frac{1}{\gamma_{Rd}}R\left(\eta \frac{X_{k}}{\gamma_{m}};a_{d}\right)$$

where: $\gamma_{Rd}$—partial factor taking into account the uncertainty of the theoretical calculation model of the structure, η—the mean value of the conversion factor taking into account: volume and scale effects, effects of moisture and temperature and any other relevant parameters, $X_{d}$—the design value of a material or product property, $a_{d}$—the design values of the geometrical data, $X_{k}$—the characteristic value of the material or product property, $\gamma_{m}$—partial factor for materials, including uncertainties about geometry and modelling.

Taking into account the dependence (4) specified in the standard [26], design load capacity can be presented in the form (5):

(5) $$R_{d}=R\left(\eta \frac{X_{k}}{\gamma_{M}};a_{d}\right)$$

Referring the presented standard provisions to the considerations that are the subject of this article—compressed masonry structures, the material parameters in Equation (5) should be taken as the characteristic and computational compressive strength of the analyzed masonry structure, i.e., a compressed solid brick pillar on standard mortar and a compressed wall made of from autoclaved aerated concrete blocks on a thin-layer mortar: *X_k_* = *f_k_*; *X_d_* = *f_d_*. The research carried out in this work was carried out on a natural scale, therefore the conversion factor was omitted in the calculations concerning the partial safety factor.

The characteristic value of the strength of the masonry structure was determined using the calculation fractile factor *k_n_* assigned to the quantile of the order of 0.05, according to Table 7 [26].

The characteristic value of the compressive strength of the masonry was determined from the formulas:

- for normal distribution
  (6) $$f_{k}=f_{cm}\left(1-k_{n}\nu_{x}\right)$$
- for logarithmic—normal distribution
  (7) $$f_{k}=\exp\left(m_{y}-k_{n}s_{x}\right)$$
  where: $f_{cm}$—mean compressive strength of masonry [MPa], $k_{n}$—characteristic fractile factor according to Table 7, $\nu_{x}$—coefficient of variation, $m_{y},s_{y}$—was determined from the Formulas (8) and (9):
  (8) $$m_{y}=1/n\;\sum ln\left(x_{i}\right)$$
  (9) $$s_{y}=\sqrt{ln\left(\nu_{x}{}^{2}+1\right)}\approx \nu_{x}$$

The design value of the compressive strength of the masonry is determined from the dependencies—Equations (10) and (11):

- for normal distribution
  (10) $$f_{d}=f_{cm}\left(1-\alpha_{R}\;\beta \nu_{x}\right)$$
- for logarithmic—normal distribution
  (11) $$f_{d}=f_{cm}\exp\left(-\alpha_{R}\;\beta \nu_{x}\right)$$
  where: $\alpha_{R}$—sensitivity factor for resistance, $\alpha_{R}=0.8\;$respectively, provided 0.16 ≤ *σ_E_*/*σ_R_* < 7.6, *β*—reliability index.

The value of the partial factor for the material properties *γ_m_* is determined from the relationship

- for normal distribution
  (12) $$\;\;\gamma_{m}=\left(1-k_{n}\nu_{x}\right)/\left(1-\alpha_{R}\;\beta \nu_{x}\right)\;$$
- for logarithmic–normal distribution
  (13) $$\gamma_{m}=\exp\left[m_{y}-k_{n}s_{y}\right]/f_{k,m}\exp\left(-\alpha_{R}\;\beta \nu_{x}\right)$$

On the basis of tests of masonry structures, determining the value of the *γ_Rd_* coefficient expressing the model error is very difficult, and there are very few analyses and tests related to the determination of the value of the partial coefficient for the load-bearing capacity, taking into account the uncertainty of the calculation model for masonry structures. The standard [26] recommends for the designed masonry structures to adopt the value of this coefficient as for reinforced concrete structures, at the level of *γ_Rd_* = 1.1. In the case of existing masonry structures, the detailed identification of which is limited, it is proposed to adopt slightly higher values, e.g., *γ_Rd_* = 1.15 [50]. In the article, an attempt was made to determine the value of *γ_Rd_* for the analyzed compressed masonry structures. For this purpose, Equation (14) was used:

(14) $$\gamma_{Rd}=\frac{R_{obs,k}}{R_{cal,k}}$$

where: $R_{obs,k}$—the characteristic value of the load capacity of the structure elements determined from the tests, $R_{cal,k}$—the characteristic load capacity of the tested structure element determined analytically on the basis of the adopted structure model as a function of the characteristic strength of the material *f_k_*, in the case of the 18 analyzed structures of the Formula (15):

(15) $$R_{cal,k}=\left(1-\frac{e_{i}}{t}\right)\cdot t\cdot l\cdot f_{k}$$

where: t, l—dimensions of the masonry structure, $e_{i}$—the eccentricity of the load transfer, the value of the initial eccentricity resulting from the imprecision of the structure performance obtained from the tests was adopted,

- for a brick pillar on general purpose mortar
  (16) $$f_{k}=0.45f_{b,k}{}^{0.7}f_{m,k}{}^{0.3}$$
- for AAC masonry wall on a thin-layer mortar
  (17) $$f_{k}=0.75f_{b,k}{}^{0.85}$$
  where: $f_{b,k}$—characteristic value of the compressive strength of the masonry unit determined using the fractile factor $k_{n}$ [MPa], $f_{m,k}$—characteristic value of the compressive strength of the mortar determined using the fractile factor $k_{n}$ [MPa].

The calculations of the *γ_m_* and *γ_Rd_* coefficients, as well as the consequent *γ_M_*, were performed for the results obtained in the experimental tests—Table 8. In the case of the compressive strength of the masonry, a 50-year reference period was adopted, assuming the RC2 reliability class and the corresponding value of the reliability index, i.e., *β* = 3.8.

The calculation of the partial safety factors for the assumption of normal distribution was also carried out in combination with examples of other experimental tests of masonry structures, the results of which were taken from the literature—Table 9. To this end, the average coefficient of variation $\nu_{Rm}$ was calculated from the given data, taking into account the coefficients of variation of the brick pillar and masonry walls from AAC that are the subject of the study. In order to determine the value of the mean coefficient of variation $\nu_{Rm}$, the central limit theorems were used.

Calculations of the partial factor for the load capacity, taking into account the literature data, were performed for RC2 reliability class, for the 50-year reference period and taking into account the normal and log-normal probability distribution of the compressive strength (Table 10). In these calculations, the standard recommended value of the *γ_Rd_* coefficient with a constant value of 1.1 was adopted.

**Figure 20:**
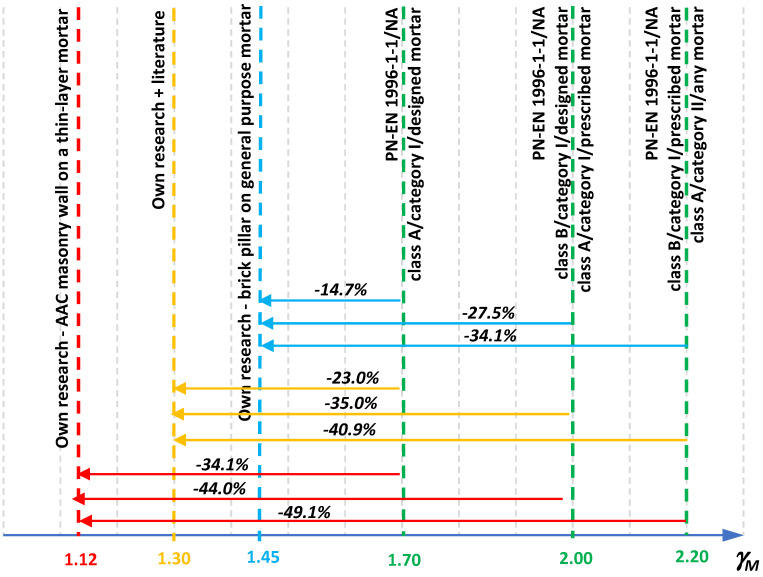
Comparison of the calculated values of the partial safety factor for masonry structures designed in the RC2 reliability class with the values recommended in the National Polish Annex of the standard [PN-EN 1996-1-1: 2010].

Based on the performed calculations, taking into account the results of own research and the data from the tests included in the literature available to the author, it was found that the obtained values of the partial coefficient *γ_M_* for the compressed masonry structures, regardless of the adopted density function (normal distributions (N), logarithmic–normal distribution (LN)), for the load capacity differ by up to 10%. The obtained difference in the value of *γ_M_* is the result of adopting a constant value of the partial factor for the load capacity, taking into account the uncertainty of the load capacity calculation model at the level of *γ_Rd_* = 1.1. The obtained values of partial factors (Table 8 and Table 10) were compared with the values of partial factors recommended in the National Polish Annex of the standard [4] (Figure 20).

The graphical list of the obtained values of partial factors for the results of own research and for the data from the available literature was made for the reliability class RC2, because for this class the safety of the structure is ensured with the use of a set of partial factors for the load capacity and applicable loads in Eurocodes.

## 5. Discussion

The real-scale experimental tests of masonry structures presented in this article are an extension of the available knowledge on the behavior of masonry structures under compressive load. In addition, information on the obtained values of coefficients of variation are a significant basis for conducting advanced probabilistic analyses of such constructions. The achieved values of coefficients of variation for masonry components proved to be satisfactory and adequate to the emerging technological progress in the process of production and incorporation of masonry components. The variability of compressive strength of basic masonry structures was used to determine safety using the probabilistic method using the recommendations of PN-EN 1990 [26]. The calculations of safety factors in the first stage were limited only to the results of the research being the subject of this study. The obtained values of the coefficients, both assuming the normal distribution and the logarithmic–normal distribution proved to be satisfactory, which is smaller than the coefficients recommended in the Polish National Annex PN-EN 1996-1-1 [4]. Reliability and safety analyses of masonry structures were also carried out taking into account the results of experimental studies available in the literature, analyzing the coefficient of variability of compressive strength of masonry. In this case, the value of the safety factor also proved to be relatively small. Based on the conducted analyses, it should be concluded that the values of partial factors recommended in the National Annex to the standard PN-EN 1996-1-1 [4] are relatively conservative. For the analyzed examples, the highest value of the partial factor was obtained for a brick pillar on a standard mortar. It was a value equal to 1.45, which indicates that the obtained value is more than 27% lower than the average value of the coefficient recommended in the standard [4].

The integration of the design process with the assessment of the reliability and safety of masonry structures in the field of estimating the value of the safety coefficients using probabilistic methods of reliability assessment may contribute to increasing the economic efficiency of the implementation of masonry structures.

## Figures and Tables

**Table 1:** Provisions concerning the characteristic compressive strength of masonry according to polish annex of the PN-EN 1996-1-1 [4].

Masonry Configuration	Algorithm for Determining the Characteristic Strength
Masonry made of any masonry units on general purpose mortar or lightweight mortar	$f_{k}=Kf_{b}{}^{0.7}f_{m}{}^{0.3}$
Masonry made of clay units of group 1 and 4, calcium silicate, aggregate ~ concrete and autoclaved aerated concrete units where *f_b_* ≥ 2.4 MPa on thin layer mortar	$f_{k}=Kf_{b}{}^{0.85}$
masonry made of autoclaved Aerated concrete units where *f_b_* < 2.4 MPa on thin layer mortar	$f_{k}=0.8Kf_{b}{}^{0.85}$
Masonry made of clay units of group 2 and 3	$f_{k}=Kf_{b}{}^{0.7}$

$f_{b}$ no more than: 75 MPa—masonry units group 1 on general purpose mortar or lightweight mortar, 50 MPa—masonry units group 1 on thin layer mortar, 35 MPa—masonry units group 2, 15 MPa—masonry units group 3 and 4. $f_{m}$ no more than: 20 MPa and 2$f_{b}$—masonry units group 1 on general purpose mortar, 20 MPa and 1$f_{b}$—masonry units group 2, 3, 4 on general purpose mortar, 10 MPa—masonry units on lightweight mortar or thin layer mortar. in the case of masonry with a longitudinal joint, values $f_{k}$ must be additionally multiplied by η=0.8.

**Table 2:** Values of partial safety factors for masonry according to PN-B-03002:1999 [20].

Masonry Production Category	Class of Execution of Works	
	A	B
I	1.7	2.2
II	2.2	2.5

**Table 3:** The relevant values of the partial factor for materials $\gamma_{M\;}$ according PN-EN 1996-1-1 [4,23].

Material	Class of Execution of Works				
	1	2(A)	3(B)	4	5
Masonry made with units of category I, designed mortar	1.5	**1.7**	**2.0**	2.2	2.5
Masonry made with units of category I, prescribed mortar	1.7	**2.0**	**2.2**	2.5	2.7
Masonry made with units of category II, any mortar	2.0	**2.2**	**2.5**	2.7	3.0

**Table 4:** Recommended minimum values for reliability index and maximum probability of destruction (ultimate limit states) (PN-EN 1990 [26]).

Reliability Class	Minimum Values for β $/\mathrm{Maximum}\;\mathrm{Probability}\;P_{f}$	
	1 Year Reference Period	50 Years Reference Period
RC3	β=5.2; $P_{f}\;≅\;9.9\times 10^{-8}$	β=4.3; $P_{f}\;≅\;8.5\times 10^{-6}$
RC2	β=4.7; $P_{f}\;≅\;1.3\times 10^{-6}$	β=3.8; $P_{f}≅\;7.1\times 10^{-5}$
RC1	β=4.2; $P_{f}\;≅\;1.2\times 10^{-5}$	β=3.3; $P_{f}\;≅\;4.8\times 10^{-4}$

**Table 5:** Coefficients of variation of compressive strength of various types of masonry estimated in the literature.

Author	Coefficients of Variation ν	Random Variable
(Holicky, Markova 2002 [32])	0.20	compressive strength
(Schueremans 2001 [33])	0.19	compressive strength
(Grauber, Glovienka 2008 [31])	0.20	compressive strength
(Brehm, Lissel 2012 [30])	0.17–0.19	compressive strength

**Table 6:** Value estimators and standard deviation of compressive strength, modulus of elasticity, and Poisson’s ratio for pillar and masonry wall models.

Model Properties		The Classic Method of Data Evaluation			Data Evaluation Immunity Method		
		Average Value	Standard Deviation	Coefficient of Variation	Median	Standard Deviation	Coefficient of Variation
Models of clay brick pillars	*f_k_* [MPa]	7.63	1.23	16.1%	8.17	1.38	16.9%
	*E_y_* [MPa]	5296	1392	26.3%	5573	2222	39.9%
	*ν_xy_*	0.27	0.04	16.1%	0.30	0.05	15.3%
Wall model of AAC	*f_k_* [MPa]	1.95	0.14	7.1%	1.97	0.22	11.0%
	*E_y_* [MPa]	1434	479	33.4%	1295	350	27.1%
	*ν_xy_*	0.21	0.04	19.2%	0.20	0.04	20.0%

**Table 7:** Values of $k_{n}$ for the 5% characteristic value (PN-EN 1990 [23,26]).

n	1	2	3	4	5	6	8	10	20	30	∞
*v*_x_ known	2.31	2.01	1.89	1.83	1.80	1.77	1.74	1.72	1.68	1.67	1.64
*v*_x_ unknown	-	-	3.37	2.63	2.33	2.18	2.00	1.92	1.76	1.73	1.64

**Table 8:** Values of partial safety factors of the analyzed masonry structures determined for the RC2 reliability class and the 50-year reference period.

Type of Masonry Material/Distribution		Partial Safety Factors		
		*γ_m_*	*γ_Rd_*	*γ_M_*
Brick pillar on general purpose mortar	N *	1.36	1.07	1.45
	LN **	1.20	1.08	1.30
AAC masonry wall on a thin-layer mortar	N *	1.12	1.00	1.12
	LN **	1.09	1.00	1.09

* normal distributions. ** logarithmic–normal distribution.

**Table 9:** Sample results of experimental tests of masonry structures presented in national literature—average masonry strength, the coefficient of variation.

No.	Type of Masonry Material	Research Author	Average Compressive Strength	Coefficient of Variation
			$f_{k,mean}\;\left[MPa\right]$	v [%]
1	Clay brick	(Drobiec i inni, 2010 [52])	9.55	5.6
2	Masonry units from AAC	(Jasiński, 2017 [53])	2.97	14.0
3	Silicate masonry units	(Jasiński, 2017 [53])	11.29	4.0

**Table 10:** Values of partial safety factors of the analyzed masonry structures determined for the RC2 reliability class and the 50-year reference period.

Type of Masonry Material/Distribution		Partial Safety Factors		
		*γ_m_*	*γ_Rd_*	*γ_M_*
Various (own research + literature)	N *	1.18	1.10	1.30
	LN **	1.14	1.10	1.25

* normal distributions. ** logarithmic–normal distribution.
